# Effects of Shugan Hewei Granule on Depressive Behavior and Protein Expression Related to Visceral Sensitivity in a Rat Model of Nonerosive Reflux Disease

**DOI:** 10.1155/2019/1505693

**Published:** 2019-01-02

**Authors:** Yi Wang, Guanwu Li, Xiaosu Wang, Shengliang Zhu

**Affiliations:** ^1^Department of Gastroenterology, Yueyang Hospital of Integrated Traditional Chinese and Western Medicine, Shanghai University of Traditional Chinese Medicine, Shanghai, China; ^2^Department of Radiology, Yueyang Hospital of Integrated Traditional Chinese and Western Medicine, Shanghai University of Traditional Chinese Medicine, Shanghai, China

## Abstract

**Objective:**

To explore the effect of Shugan Hewei Granule (SGHWG) and to provide the experimental basis for its clinical application.

**Methods:**

40 healthy male Wistar rats were divided into 5 groups, with 8 rats in each group, including control group, model group, normal saline (NS) group, SGHWG group, and Rabeprazole group. The control group was not treated. The model group was treated with fructose intake and mental stress to be the model of NERD. The other groups were treated as the model group and then gavaged with the corresponding drugs. The pH value of lower third of esophagus, immobile time in tail suspension test, CRF protein expression in both hypothalamus and anterior cingulate cortex (ACC), and SP protein in esophageal mucosa in lower third of esophagus detected by immunofluorescence and NMDAR1 protein expression in spinal cord detected by immunohistochemistry of each group were compared.

**Results:**

The pH values of both the SGHWG group and the Rabeprazole group were higher than that of the model group (*P*<0.01), but the Rabeprazole group increased more obviously. The immobile time of the SGHWG group was shorter than that of the model group (*P*<0.01) and the Rabeprazole group (*P*<0.05). The expression of the CRF in the hypothalamus and ACC, NMDAR1 in the spinal cord, and SP in the esophageal mucosa in lower third of esophagus of the SGHWG group decreased significantly, compared with the model group (*P*<0.01), and was obviously lower than that in the Rabeprazole group (*P*<0.05).

**Conclusions:**

This study provided an evidence that SGHW formula was inferior to Rabeprazole in acid inhibition, but it might reduce the expression of CRF protein of hypothalamus and ACC, lower the levels of NMDAR1 in spinal dorsal horn and SP in esophageal mucosa in lower third of esophagus, and regulate depressive behavior simultaneously, related to the improvement of visceral hypersensitivity in rat model of NERD.

## 1. Background

As a subtype of gastroesophageal reflux disease (GERD), nonerosive reflux disease (NERD) is a disease, with which patients suffer reflux-related symptoms caused by gastric contents inflowing into esophagus, but lack the endoscopic mucosal damage of esophagus [1], and it accounts for 50% to 70% [2] of GERD. The pathogenesis of NERD is complicated and has not been quite clear. Proton pump inhibitor (PPI) is often ineffective because of most NERD patients' low life satisfaction, poor sleep quality, and functional esophageal disorders [3, 4]. With deep insight into NERD, mental state, visceral sensitivity, and the relationship between them have been paid more and more attention in the occurrence of NERD. Studies have shown that negative mental state can cause visceral hypersensitivity through the brain-gut axis, and the patients' esophageal hypersensitivity is related to the sensitization of nerve endings, spinal cord, and cerebral center [5–7]. Corticotrophin releasing factor (CRF), N-methyl-D-aspartate receptor (NMDA-R), and substance P (SP) may participate in esophageal sensitization in the brain, spinal cord dorsal horn, and esophagus in turn.

The specialty of GERD in our hospital, as the “12th Five-Year” key specialty of the State Administration of Traditional Chinese Medicine, has devoted to the clinical and experimental studies of NERD for a long time and found Shugan Hewei Granule (SGHWG) made a good effect on NERD patients. In order to clarify the relationship between NERD and emotion and the mechanism of action of SGHWG on NERD, a NERD rat model was established on the basis of prestudy and preliminary experiment. The CRF protein of the hypothalamus and the anterior cingulate cortex (ACC), the NMDAR1 protein of the spinal cord, the SP protein expression in the mucosa of peripheral esophagus, and the suspension time of the rat model were detected to explore the effect of SGHWG and to provide the experimental basis for its clinical application.

## 2. Materials and Methods

### 2.1. Experimental Animals

40 healthy male Wistar rats, aged 6 weeks, weighing 200±20 grams, clean grade, were supplied by Shanghai Slack Laboratory Animal Co., Ltd. They were maintained at 20 ± 2°C, 50 ± 10% relative humidity, under a 12-h light: 12-h dark cycle, and their padding had been sterilized by high pressure. The rats had free access to tap water and a normal standard chow diet and they would be housed in these facilities for at least 1 week before the experiment.

### 2.2. Drugs

The SGHWG was made of inula terrier, reddle, rhizoma coptidis, evodia rutaecarpa, ginger, calcined concha arcae, radix bupleuri, rhizoma corydalis, stir-baked fructus gardeniae, fructus aurantii, rhizoma polygonati, and radix liquiritiae and was produced by Jiangyin Tianjing Pharmaceutical Co., Ltd. The ratio of conversion of the drug dosage between rat and human was 6.3 [8], so the amount of the granules needed was 11.07g/kg·d. The granules were mixed into the solution and stored in the refrigerator at 4°C before use. The required dose of Rabeprazole sodium enteric-coated tablets, produced by Lunan Beite Pharmaceutical Co., Ltd., was 1.80mg/kg·d according to the same ratio of conversion. The tablets were dissolved in the deionized water and stored at 4°C.

### 2.3. Reagents and Experimental Apparatus

Reagents: anti-NMDAR1 antibody (rabbit polyclonal antibody to NMDAR1 ab52177, Lot: GR94962-8), Anti-Corticotropin Releasing Factor antibody (Rabbit polyclonal antibody to Corticotropin Releasing Factor ab8901, Lot: GR3186-34), and anti-substance P antibody (Rabbit polyclonal antibody to substance P ab216414, Lot: GR300475-1) were supplied by Abcam, UK. Biotinylated goat anti-rabbit IgG, goat anti-rabbit IgG-FITC, and goat serum (raw fluid) were supplied by Suo Lai Bao Technology Co., Ltd., Beijing, China. DAB color reagent kit (DAB-2031) was supplied by Maxin Biotechnology Development Co., Ltd., Fuzhou, China. D-fructose was supplied by Mclean Biochemical Technology Co., Ltd., Shanghai, China. Polyformaldehyde and chloral hydrate were supplied by Shan Pu Chemical Co., Ltd., Shanghai, China. Xylene and anhydrous ethanol were supplied by Qiang Shen Functional Chemical Co., Ltd., Jiangsu, China.

Experimental apparatus: 24-hour esophageal pH-impedance monitor (HSYS-REC-LT2) and measurement catheter (REF MMS G-88487, Lot: 1762647A) were provided by Medical Measurement Systems B.V., Netherlands. High speed freezer centrifuge 3-18K was provided by Sigma, Germany. Rocking bed for decolorization ZD-9550 and oscillator were provided by Qilin Beier Instrument Manufacturing Co., Ltd., Jiangsu, China. Constant temperature magnetic agitator was provided by Hong Yu Science Instrument Factory, Jiangsu, China. Electronic balance ME3002T/02 was provided by Mettle Toledo Instrument Co., Ltd., Shanghai, China. Water insulation-constant temperature incubator GSP-9160MBE was provided by Bo Xun Bioinstrument Co., Ltd., Shanghai, China. Blood glucose meter was provided by Bayer HealthCare LLC, Germany. Leica microscope DM2700P, LAS V4.0, and Leica QWin V3 image analysis software were provided by Leica, Germany. Image-Pro Plus 6.0 image analysis software was provided by Media Cybernetics, USA.

### 2.4. Animal Grouping, Model Establishment, and Drug Administration

40 Wistar rats were randomly divided into 5 groups: control group, model group, normal saline (NS) group, SGHWG group, and Rabeprazole group and every 8 rats were in one group.

The control group was not treated. The model group used the method of Zayachkivska and Mizoguchi [9, 10] to treat the rats with fructose intake and mental stress. They were given free fructose water (200g/L), placed in restraint cages, and immersed vertically to the level of the xiphoid process in a water bath of 22 ± 2°C for 2 hours a day for continuous 28 days. The NS group, SGHWG group, and Rabeprazole group were treated as the model group (both fructose intake and mental stress) and then gavaged with the corresponding drugs from the 15th to the 28th day of the experiment, 2 times a day, 1ml per 100g in weight each time.

### 2.5. Sample Collection

After 10% chloral hydrate (0.3ml per 100g in weight) for deep anaesthesia, the rats were opened the chests and were inserted catheters through their left ventricles to the ascending aortas. We cut their right auricles, and at the same time 0.9% NS solution about 200 ml was used to rinse the blood of their whole bodies by pressured perfusion until their livers were completely white and the NS solution outflowed from their right auricles was colorless. The precooling 4% polyformaldehyde fixed solution (pH 7.4) 500 ml was used for enhancing perfusion for about one hour until their limbs and spinal cords became hard and then we took their spinal segments (T1-T6), the whole brain, and the lower third of esophagus (from 15mm above and 2mm below the esophageal sphincter) into paraformaldehyde for fixation. After embedded by paraffin, the above tissues would be continuously sliced from coronary and cross section (40*μ*m per slice) according to the anatomical location of histologic atlas of Gartner and Hiatt [11] before hematoxylin and eosin, immunohistochemistry, and immunofluorescence staining.

### 2.6. Testing Indexes and Methods

#### 2.6.1. Test of pH Value in the Lower Third of the Esophagus

Before measurement, all rats had fasted for 12 hours. When the rats were under deep anaesthesia with 10% chloral hydrate (0.3ml per 100g in weight), the electrode of the pH value recorder was placed at 1 cm above gastroesophageal junction, and after 1min, the instantaneous pH values of the lower third of esophagus of the rats were recorded.

#### 2.6.2. Observation of Immobile Time in Tail Suspension Test

When all rats had fasted for 12 hours on the 29^th^ day of the whole experiment, the rats were fixed at about 3cm from the end of the tail for suspension in a quiet environment. Their inverted head were about 20cm fom the ground. When they were displayed as a passive state and their limbs' movements were disappeared, the rats were regarded as immobility. For each rat, a total of 6 min was observed, and the cumulative time of the immobile state of the rats within the later 4 min was recorded [12].

#### 2.6.3. HE Staining of Esophageal Mucosa in Lower Third of Esophagus

(1) Paraffin sections were dewaxed to water; (2) all sections were stained by hematoxylin for 5 min; (3) all sections were cleaned by running water for 10 min; (4) all sections were stained by 0.5% eosin for 1-3 min; (5) all sections were cleaned by distilled water for 30 sec; (6) stained sections were dehydrated by pure alcohol and then were made transparent by xylene; (7) the sections were sealed by neutral gum and then were observed under light microscope. The grading injury indexes of esophageal mucosa were evaluated according to Esohisto guidance [13].

#### 2.6.4. Detection of the Expression of CRF in the Hypothalamus and ACC and SP in the Esophageal Mucosa in Lower Third of Esophagus by Immunofluorescence FITC

(1) Paraffin sections were made dewax to water; (2) PBST liquid was used to clean each section for 10min; (3) all sections were soaked in 3%H_2_0_2_ for 15min, which were protected from light to inactivate endogenous peroxidase; (4) PBST liquid was used to clean each section for 30min; (5) citrate was used to repair the antigen for 30min, temperature of water kept between 92~99°C; (6) PBST liquid was used to clean each section for 30min; (7) all sections were soaked in 0.1% Triton X-100 for 10min to increase the permeability of the membrane; (8) PBST liquid was used to clean each section for 30min; (9) after drying the slices, we dripped the 10% goat serum on the sections and then incubated them at room temperature for 60min; (10) all the slices were dried again and were incubated with primary antibody (1/200 in diluent) overnight at 4°C; (11) taken out of the fridge, each piece was first washed in PBST for discontinuation of reaction and then was cleaned in PBST liquid for 30min; (12) after drying the slices, we dripped the IgG-FITC of sheep anti-rabbit and then incubated them at room temperature for 60min; (13) PBST liquid was used to clean each section for 30min; (14) after dried, the slices were dripped with DAPI; (15) we used glycerin gelatin for seal and then observed them under fluorescence microscope. The immunofluorescence positive products were light green, obvious green, or bright green with dazzling fluorescence under fluorescence microscopy and were analyzed by image analysis software. We chose one slice for each sample, and randomly observed 5 views for the expression of CRF in the hypothalamus and ACC and SP in the esophageal mucosa in lower third of esophagus under the high magnification microscope (10*∗*40). Each integrated optical density (IOD) of the positive reaction site was recorded and the average value of IOD in the 5 fields was calculated to represent the quantity of antigen expression. The larger the quantity, the stronger the positive expression.

#### 2.6.5. Detection of Expression of NMDAR1 Protein in Spinal Cord by Immunohistochemical SP Method

(1) ~ (11) were the same as immunofluorescence FITC. (12) After drying the slices, we dripped the second antibody on the sections and then incubated them at room temperature of 37°C for 30 min; (13) PBST liquid was used to clean each section for 30min; (14) after drying the slices, we dripped HRP on the sections and then incubated them at room temperature of 37°C for 30min; (15) PBST liquid was used to clean each section for 30min; (16) DAB solution was used for coloration protected from light. When they showed brown, we washed the slices in pure water; (17) all the slices were rinsed by water for 10min; (18) hematoxylin was used for staining for 10sec; (19) all the slices were rinsed by water for 7-10min; (20) all the slices were dehydrated, sealed by neutral balsam, and observed under light microscope. The positive products of the immunohistochemical SP method which showed thin yellow fine particles, brown yellow particles, and brown yellow coarse granules were analyzed with image analysis software under optical microscope. One slice was selected to represent each specimen and the expression of NMDAR1 in the spinal cord of 5 fields of vision at random was observed under the high magnification microscope (10*∗*40). The average optical density (AOD), meaning IOD/area, of the positive reaction site of each field was calculated. The average value of AOD in 5 fields demonstrated the quantity of antigen. The larger the value, the more the antigen expressed.

### 2.7. Statistical Analysis

SPSS 18.0 statistical package (provided by IBM, USA) was used for data entry and processing. The data which accorded with normal distribution was expressed as mean ± standard deviation (x ± s) or as median (four quantile range), that is, M (P25, P75). When the data accorded with normal distribution and homogeneity of variance, one-way analysis of variance (ANOVA) and LSD-t test were used for the intergroup comparisons, or nonparametric test was used. For normality and homogeneity of variance tests, a statistical difference was defined as* P*<0.1, but for other tests a statistical difference was defined as* P*<0.05 and a statistically significant difference was defined as* P*<0.01.

## 3. Results

### 3.1. Comparison of Body Weight and Blood Glucose of Rats between the Model Group and the Control Group

The blood glucose levels of rats between the model group and the control group were statistically different at the end of the second and fourth week, and the former was higher (*P*<0.05). The weights of rats between the two groups were statistically significant at the end of the fourth week, and the former was heavier (*P*<0.05, Table 1).

### 3.2. Comparison of the Pathological Changes of the Esophageal Mucosa in Lower Third of Esophagus of Rats between the Model Group and the Control Group

**Figure 1:**
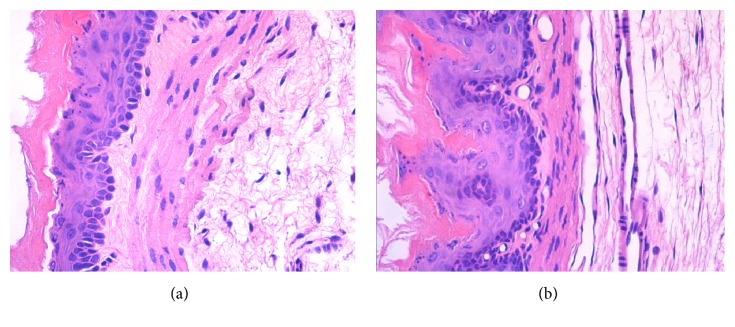
**Pathological changes of the esophageal mucosa in lower third of esophagus of rats (HE staining, ×400)**. Notes: (a) control group; (b) model group (HE staining, ×400).

After the esophagus was cut longitudinally, the esophageal mucosa in lower third of esophagus of both the control group and the model group looked undamaged. After paraffin embedding and HE staining, the rats' esophageal mucosa in lower third of esophagus of the model group showed basal cell layer hyperplasia, papillary elongation, dilated intercellular spaces, increases of intraepithelial eosinophils, neutrophils, and mononuclear cells under light microscope. Almost no significant pathological changes were observed in the structure of the esophageal wall in the control group (Table 2, Figure 1).

### 3.3. Comparison of pH Values of the Lower Third of Esophagus of Rats in Each Group

**Figure 2:**
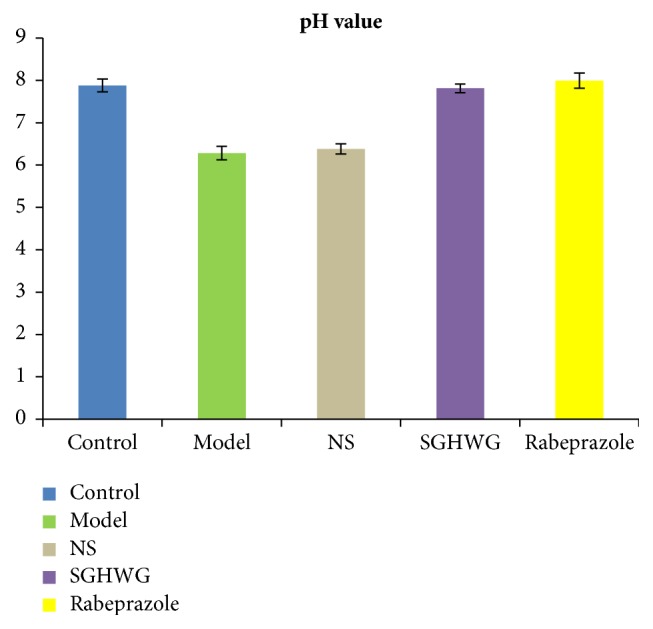
pH value of the lower third of esophagus of rats.

The pH value of the lower third of esophagus of the model group was lower than that of the control group (*P*<0.01), indicating that there was a pathological acid reflux in the model group. There was no significant difference between the NS group and the model group (*P*>0.05). The pH values of both the SGHWG group and the Rabeprazole group were higher than that of the model group (*P*<0.01), but the pH value of the Rabeprazole group increased more obviously (Table 3, Figure 2).

### 3.4. Comparison of Immobile Time of Rats in Tail Suspension Test in Each Group

**Figure 3:**
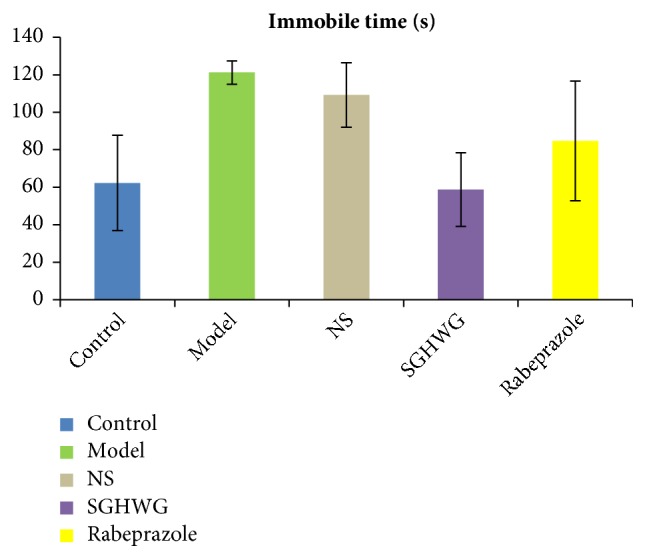
Immobile time of rats in tail suspension test.

The immobile time of rats in the model group was significantly longer than that in the control group (*P*<0.01), indicating that the rats of model group had depressive behavior. Compared with the model group, the NS group showed no difference (*P* > 0.05). The immobile time of both the SGHWG group and the Rabeprazole group was shorter than that of the model group (*P*<0.01), but the SGHWG group shortened more obviously (*P*<0.05, Table 4, Figure 3).

### 3.5. Comparison of the Expression of CRF Protein in Hypothalamus and ACC of Rats in Each Group

**Figure 4:**
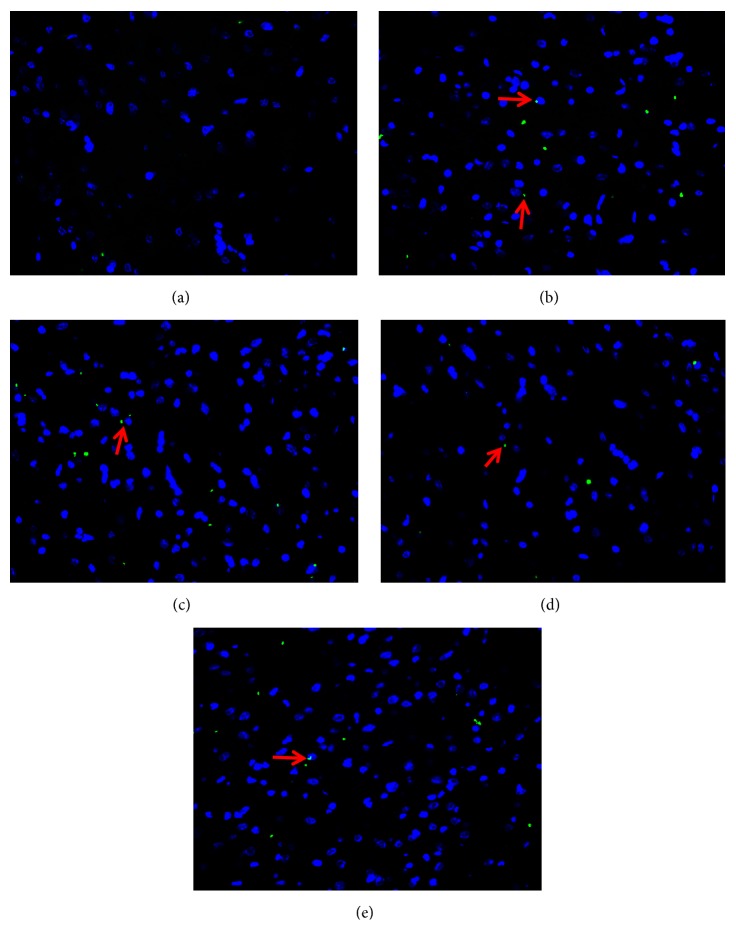
**Expression of CRF protein in hypothalamus of rats**. Notes: (a) control group, (b) model group, (c) NS group, (d) SGHWG group, and (e) Rabeprazole group (immunofluorescence staining, ×400).

**Figure 5:**
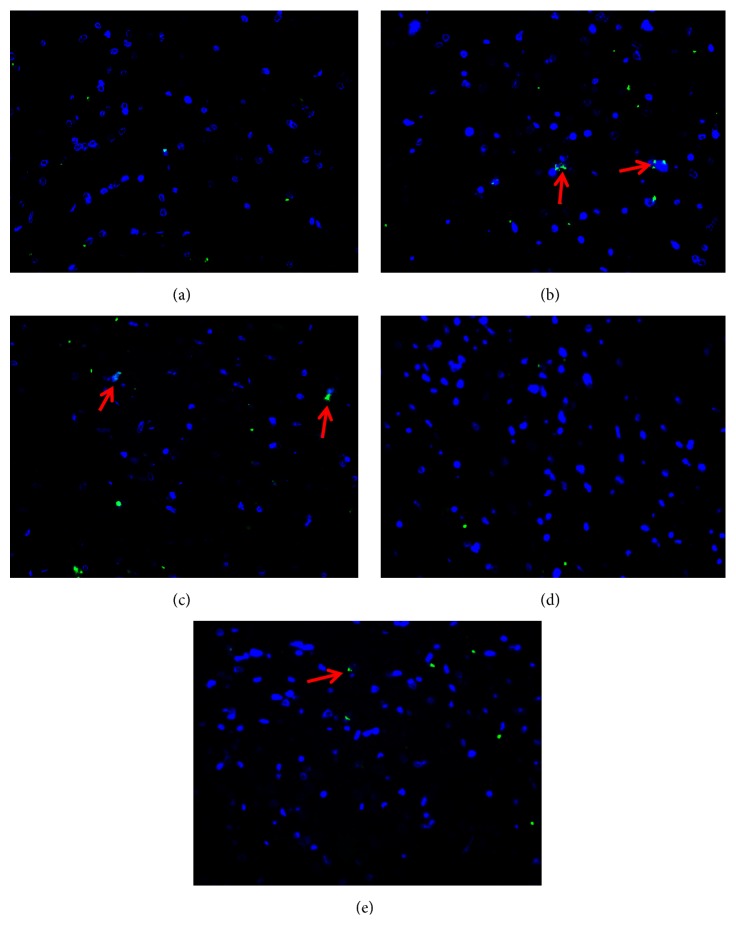
**Expression of CRF protein in anterior cingulate cortex of rats**. Notes: (a) control group, (b) model group, (c) NS group, (d) SGHWG group, and (e) Rabeprazole group (immunofluorescence staining, ×400).

CRF was expressed in the hypothalamus and ACC of rats in each group, of which green immunofluorescent product was mainly distributed in the cytoplasm of neurons. The shapes of neurons in these area were mostly round, spindle, and elliptical. As shown in Figures 4 and 5, the immunofluorescent positive product of CRF was very little and scattered in hypothalamus and ACC of rats in the control group. The positive cells presenting as fluorescently labeled in the model group were counted more intensive than those in the control group and green of positive product of the model group was deeper. Corresponding to the result obtained in immunofluorescence assay, Table 5 showed the relative protein expression level of CRF measured by value of IOD. IOD value of the model group was more than twice as much as that of the control group (*P*<0.01). Compared with the model group, neither the NS group nor the Rabeprazole group showed any significant difference (*P*>0.05). The expression of the CRF protein in the SGHWG group significantly decreased, compared with the model group (*P*<0.01), and was obviously lower than that in the Rabeprazole group (*P*<0.05, Table 5 and Figures 4 and 5).

### 3.6. Comparison of the Expression of NMDAR1 Protein in Spinal Cord of Rats in Each Group

**Figure 6:**
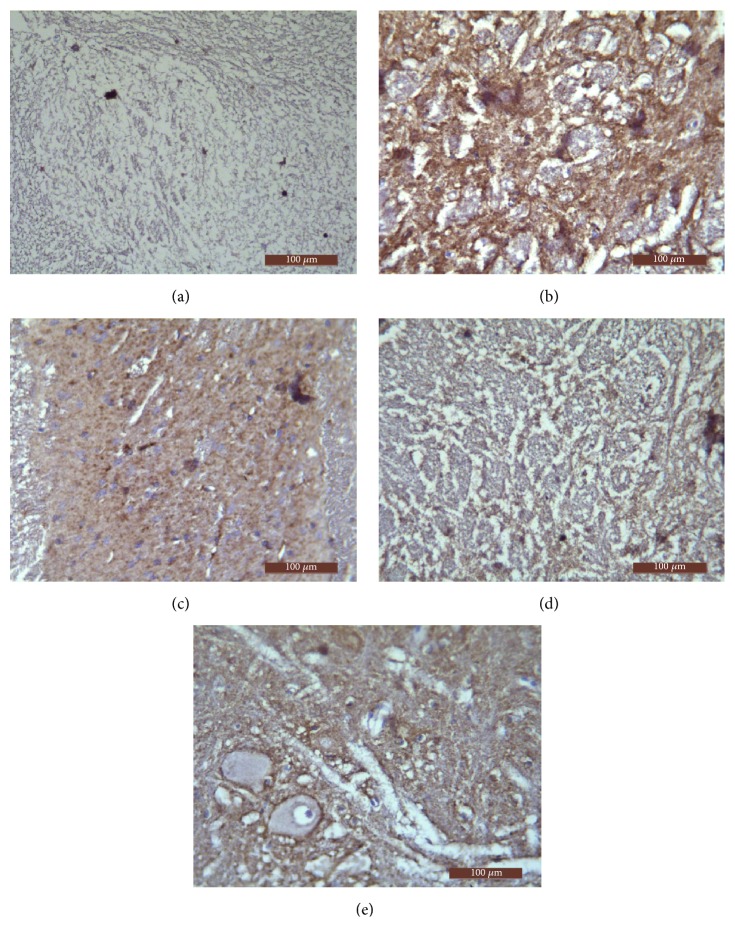
**Expression of NMDAR1 protein in spinal cord of rats**. Notes: (a) control group, (b) model group, (c) NS group, (d) SGHWG group, and (e) Rabeprazole group (immunohistochemical staining, ×400).

NMDAR1 was expressed in the posterior horn of spinal cord of rats in each group, of which brown immunopositive product was mainly distributed in the cell membrane and cytoplasm of neurons. The shapes of neurons in the spinal cord were mostly fusiform and polygonal. As shown in Figure 6, the immunohistochemical positive product of NMDAR1 was very little and scattered in spinal cord of rats of the control group. The positive cells presenting as DAB-colored in the model group were counted more intensive than those in the control group and brown of positive product of the model group was deeper. Corresponding to the result obtained in immunohistochemistry assay, Table 6 showed the relative protein expression level of NMDAR1 measured by value of AOD. The expression level of NMDAR1 protein in the spinal cord of the model group was significantly higher than that in the control group (*P*<0.01). Compared with the model group, neither the NS group nor the Rabeprazole group showed any significant difference (*P*>0.05). The expression of NMDAR1 protein in the spinal cord of the SGHWG group significantly decreased, compared with the model group (*P*<0.01), and was obviously lower than that in the Rabeprazole group (*P*<0.05, Table 6 and Figure 6).

### 3.7. Comparison of SP Protein Expression in the Esophageal Mucosa in Lower Third of Esophagus of Rats in Each Group

**Figure 7:**
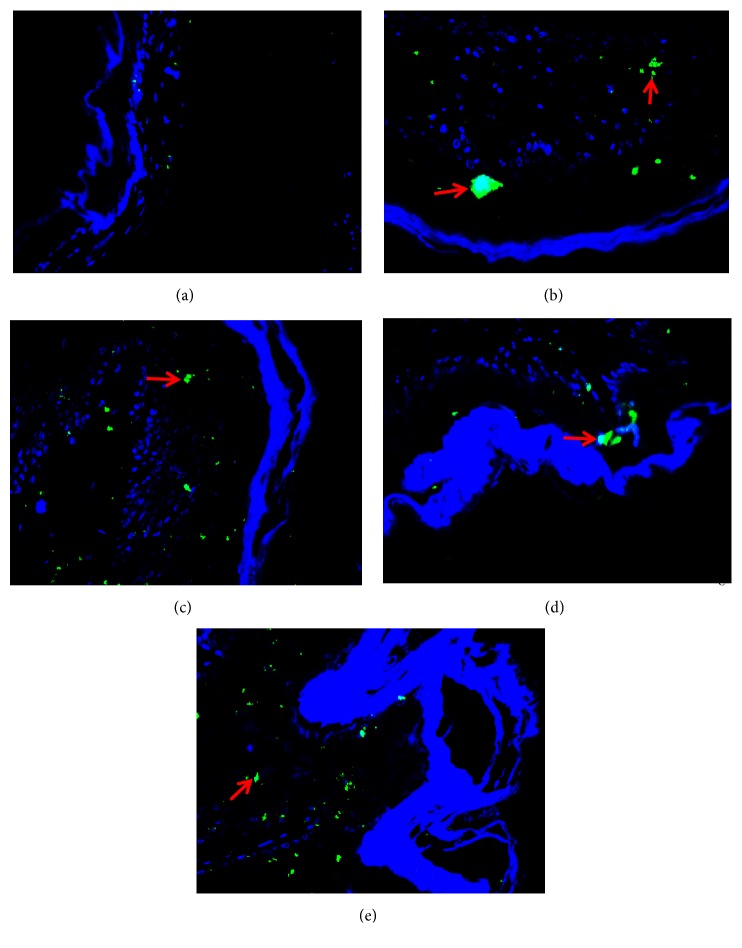
**Expression of SP protein in esophageal mucosa in lower third of esophagus of rats**. Notes: (a) control group, (b) model group, (c) NS group, (d) SGHWG group, and (e) Rabeprazole group (immunohistochemical staining, ×400).

SP was expressed in the lower third of esophagus of rats in each group, of which green immunofluorescent product was mainly distributed in the sensory nerve fibers in esophageal mucosa. As shown in Figure 7, the positive immunofluorescent product of SP was very little and scattered in esophageal mucosa of rats of the control group. The positive cells presenting as fluorescently labeled in the model group were counted more intensive than those in the control group and green of positive product of the model group was deeper. Corresponding to the result obtained in immunofluorescence assay, Table 7 showed the relative protein expression level of SP measured by value of IOD. IOD value of the model group was more than four times as much as that of the control group (*P*<0.01). There was no statistically significant difference between the NS group and the model group (*P*>0.05). The expression of SP in the esophageal mucosa in lower third of esophagus of both the SGHWG group and the Rabeprazole group decreased significantly, compared with the model group (*P*<0.05), but the decrease in the SGHWG group was more obvious (*P*<0.05, Table 7, Figure 7).

## 4. Discussion

The pathogenesis of NERD includes high viscera sensitivity of esophagus, reflux stimulation, abnormal esophageal motility, and psychological factors. However, there is no theory that can fully explain the occurrence of NERD. Therefore, most scholars believe that NERD is heterogeneous diseases and may be the result of a variety of pathogenic factors. Zhao et al. [14] found that, after chronic restraint stress, the rats' esophageal epithelial barrier function would suffer damage, which was similar to the morphological changes produced by acid instillation into the esophagus, and the acid reperfusion would aggravate the damage of the mucosa. Zayachkivska et al. [9] used fructose intake and water-immersion stress stimulation to establish the animal model of NERD. The manifestation of the esophageal mucosa of the rats was consistent with the patients with NERD, indicating that the application of mental stimulation could simulate the pathophysiological status of the patients with NERD well. In recent years, the roles of psychosocial factors and visceral hypersensitivity in the pathogenesis of NERD have attracted more and more attention.

In clinic, the incidences of anxiety, depression, sleep disorder, and autonomic nervous dysfunction were higher in patients with NERD than that in patients with erosive esophagitis (EE),another subtype of GERD, according to Pogromov [15]. Zhang et al. [16] found that 71% of NERD patients had mental and psychological abnormalities, higher than that of EE patients (57.3%), and the quality of life of NERD patients was lower than that of EE patients, which was likely to be related to the abnormal mental state of NERD patients. A large sample and prospective study conducted by Lee et al. [17] showed that neuroticism and psychological abnormality were the risk factors of NERD. Liu et al. [18] used the Symptom Checklist-90 (SCL-90) to make a psychological assessment of NERD patients in outpatient clinic. The results showed that the scores of somatization, compulsion, anxiety, paranoia, depression, and hostility in NERD patients were significantly higher than those of the normal control group, and the patients with pathological acid reflux had more mental disorders. Other studies also showed that noncardiac chest pain as a common symptom of NERD was relevant to anxiety and depression [19]. Yamasaki et al. emphasized reflux hypersensitivity affected primarily young to middle aged women and was often associated with some type of psychological comorbidity [20]. Avidan et al. [21] found that the frequency of heartburn in patients with mental disorders was higher than that in the general population, suggesting that psychological problems were likely to make reflux symptoms more perceived. Phillips et al. used functional magnetic resonance imaging (fMRI) to monitor the central nervous response of a healthy person with a phasic and painless stimulation of his esophagus, and he found that the activation of the right side of insula and the double dorsal ACC related to esophageal sensation was obviously stronger when one had negative emotions such as fear and unease than when one was calm [22]. Some researches have confirmed that psychosocial factors can induce visceral hypersensitivity through brain-gut axis [23].

Corticotropin releasing factor (CRF), which consists of 41 amino acid residues, plays an important role in the regulation of mental stress of the central nervous system. Mental stress can stimulate the release of CRF in the paraventricular nucleus (PVN) and the marginal system (including cingulate gyrus, etc.), activating the brainstem locus coeruleus to excite and facilitate the visceral senses of animals [24]. CRF released from the hypothalamus and its related brain regions is considered to be one of the important mechanisms of visceral hypersensitivity [25, 26]. At the same time, the release of CRF will activate the hypothalamic pituitary adrenal axis (HPA) to regulate the anxious behavior and make the visceral sensation closely interact with the emotion. Negative emotion can lead to the release of CRF in the hypothalamus and other related brain regions, acting on the medullary vagus dorsal nucleus, nucleus ambiguus, and spinal cord to regulate the endocrine, autonomic, immune, and behavioral responses and affect gastrointestinal motility and sensitivity [27]. Studies have shown that after mechanical and chemical stimulations activate the visceral receptors, the signal will be transferred into the posterior horn of the spinal cord and then glutamic acid releases to upregulate the N-methyl-D-aspartic acid receptor (NMDA-R) as one of the ionic excitatory amino acid receptors [28], causing an activity-dependent increase in the reactive activity of the dorsal horn neurons and a change of the neural plasticity. The reactivities of the spinal dorsal horn nerve cells to the original low-threshold stimulation and the existing afferent impulses increase. The activation threshold of the new impulses is reduced and the receptive field is enlarged, so that responses of nerves to the normal stimulation intensity are enhanced. However, after the elimination of the peripheral stimulation, the plasticity of neurons can make the spinal dorsal horn remain in high sensitive state [29–31]. As the effect of NMDA and its receptors in irritable bowel syndrome (IBS) has been confirmed, it is presumed that the NMDA receptor may join in the process of esophageal sensitization at the level of the dorsal horn of the spinal cord. SP is a peptide composed of 11 amino acids, which is a double-distributed brain-gut peptide and plays an important role in mediating visceral nociception. SP is widely distributed in capsaicin-sensitive sensory nerve C fibers [32]. It is a neuropeptide that transmits nociceptive information and an important indicator of pain. In NERD, immunohistochemistry has confirmed that OD value of SP positive products of sphincter in lower part of esophagus are negatively correlated with the initial threshold of esophageal perception and the maximum threshold of tolerated pain. Mental and physical stimulation can make nerve center, as hypothalamus, limbic brain, locus coeruleus, etc., release CRF which will combine with the receptor of peripheral sensory neurons to promote the secretion of SP, to affect the motility and secretory function of the gastrointestinal tract and the visceral sensitivity in the periphery [27].

In traditional Chinese medicine, the basic pathogenesis of NERD is that liver lose its function of dispersion and stomach loses its function of descent, leading to adverse rising of gastric Qi. Although the location of disease is esophagus and stomach, NERD is closely related to liver, gallbladder, spleen, and lung. Clinically, we used SGHW granule to treat patients with NERD and obtained good curative effect. SGHWG was made up of 9 g of Inula terrier, 15 g of reddle, 3 g of rhizoma coptidis, 3 g of evodia rutaecarpa, 3 g of ginger, 30 g of calcined concha arcae, 9 g of radix bupleuri, 9 g of rhizoma corydalis, 9 g of stir-baked fructus gardeniae, 12 g of fructus aurantii, 15 g of rhizoma polygonati, and 6g of radix liquiritiae. The prescription of SGHW complies with the pathogenesis of NERD to sooth liver and harmonize stomach by the combination of spicy and bitter herbs, cold, and warmth herbs, regulating Qi and blood, elimination, and supplement, against symptoms and pathogeny, so that gastric Qi go down not up, the liver and gallbladder keep balance not to traverse, surface and interior harmonize, ascent and descent cooperate well, and the body will be self-healing. Modern pharmacological studies [33, 34] showed that saikosaponin and ethanol extract of fructus aurantii could play an antidepressant role, and berberine could reduce visceral hypersensitivity in IBS rats. Therefore, according to the theory of traditional Chinese medicine and modern pharmacology, it is well-grounded that SGHW prescription can play a role in improving the depressive behavior and visceral hypersensitivity of NERD patients.

In this study, we found that the immobile time of the tail suspension in the model group was significantly longer than that in the control group and meanwhile model group suffered the pathological acid reflux, indicating making use of mental stimulation could simulate the pathophysiological state of NERD well. At the same time, the expressions of CRF protein in hypothalamus and ACC, NMDAR1 protein in spinal cord, and SP protein in esophageal mucosa in lower third of esophagus of model group were significantly higher than that of the control group, indicating that visceral hypersensitivity and depressive behavior coexisted in the model group. The pH values of lower third of esophagus in SGHWG group and Rabeprazole group were both higher than that in the model group, but the pH value of Rabeprazole group increased more obviously, which indicated that both SGHWG and Rabeprazole could inhibit gastric acid but Rabeprazole had stronger inhibiting capacity. The immobile time of SGHWG group and Rabeprazole group was both shorter than that of the model group, and the immobile time of SGHWG group shortened more, so we thought SGHWG could relieve the behavioral despair of NERD rats better. The expression of CRF protein in hypothalamus and ACC, NMDAR1 protein in spinal cord, and SP protein in esophageal mucosa in lower third of esophagus of SGHWG group was significantly lower than that of model group and Rabeprazole group, which suggested SGHWG might improve depression and alleviate behavioral hopelessness in NERD rats by reducing the expression of CRF protein in hypothalamus and ACC to adjust the sensitization process of visceral hypersensitive in brain; SGHWG might improve the sensitization of the spinal cord in NERD rats by reducing the expression of NMDAR1 protein in it; regulating the expression of SP protein in the esophageal mucosa in lower third of esophagus might be the intervention mechanism of SGHWG on the peripheral level of rat model of NERD.

More and more researchers have been conscious that alternative medicine techniques as herbal medicine continue to show promise, especially in NERD patients who failed antireflux treatment [35, 36]. In the future, based on the clinical efficacy of SGHWG, more systematic and scientific studies should be designed for clarifying the therapeutic mechanism of this prescription in regulating mood and visceral hypersensitivity in patients with NERD.

## 5. Conclusions

Although there existed some limitations of our research, it also provided an evidence that SGHW formula was inferior to Rabeprazole in acid inhibition, but it might reduce the expression of CRF protein of hypothalamus and ACC, lower the levels of NMDAR1 protein in spinal dorsal horn and SP protein in esophageal mucosa in lower third of esophagus, and regulate depressive behavior simultaneously, related with the improvement of visceral hypersensitivity in rat model of NERD.

## Figures and Tables

**Table 1:** Weight and blood glucose of rats in the model group and the control group $\left(\overset{-}{x}\pm s\right)$.

Group	Index	0^th^ day	14^th^ day	28^th^ day
Control	weight (g)	256.25 ± 7.30	316.88 ± 6.37	331.63 ± 10.25
	blood glucose (mmol/L)	5.40 ± 0.35	6.04 ± 0.66	5.39 ± 0.32
Model	weight(g)	251.25 ± 9.04#	321.13 ± 13.81#	350.88 ± 19.54*∗*
	blood glucose (mmol/L)	5.58 ± 0.41#	8.15 ± 2.06*∗*	6.40 ± 0.76*∗*

Notes: *∗P*<0.05; #*P*>0.05, compared with the control group.

**Table 2:** Histologic severity score of esophageal mucosa of rats in the model group and the control group $\left(\overset{-}{x}\pm s\right)$.

Group	n	Severity score
Control	8	1.50 ± 0.93
Model	8	3.63 ± 0.92*∗*

Notes: *∗P*<0.05, compared with the control group.

**Table 3:** pH value of the lower third of esophagus of rats in each group $\left(\overset{-}{x}\pm s\right)$.

Group	n	pH value of the lower third of esophagus
Control	8	7.88 ± 0.15
Model	8	6.28 ± 0.16*∗*
NS	8	6.38 ± 0.12#
SGHWG	8	7.81 ± 0.10▲
Rabeprazole	8	7.99 ± 0.18▲◊

Notes: *∗P*<0.001, compared with the control group; ▲*P*<0.001 and #*P*>0.05, compared with the model group; ◊*P*<0.05, compared with the SGHWG group.

**Table 4:** Immobile time of rats in tail suspension test in each group.

Group	n	Immobile time (s)
Control	8	62.25 ± 25.42
Model	8	121.25 ± 6.18*∗*
NS	8	109.25 ± 17.12#
SGHWG	8	58.75 ± 19.69▲◊
Rabeprazole	8	84.75 ± 31.98▲

Notes: *∗P*<0.01, compared with the control group; ▲*P*<0.01 and #*P* > 0.05, compared with the model group; ◊*P*<0.05, compared with Rabeprazole group.

**Table 5:** IOD values of CRF immunoreactive products in hypothalamus and anterior cingulate cortex of rats in each group $\left(\overset{-}{x}\pm s\right)$.

Group	n	IOD	
		Hypothalamus	Anterior cingulate cortex
Control	8	448.83 ± 357.92	730.12 ± 90.14
Model	8	1161.15 ± 740.35*∗*	1610.40 ± 628.64*∗*
NS	8	1012.25 ± 297.11#	1302.85 ± 701.37#
SGHWG	8	517.82 ± 225.40▲◊	551.05 ± 138.45▲◊
Rabeprazole	8	960.28 ± 355.07#	1391.70 ± 947.52#

Notes: *∗P*<0.01, compared with the control group; ▲*P*<0.01 and #*P* > 0.05, compared with the model group; ◊*P*<0.05, compared with Rabeprazole group.

**Table 6:** AOD values of NMDAR1 immunoreactive products in spinal cord of rats in each group $\left(\overset{-}{x}\pm s\right)$.

Group	n	AOD
Control	8	0.11 ± 0.08
Model	8	0.26 ± 0.07*∗*
NS	8	0.23 ± 0.12#
SGHWG	8	0.12 ± 0.04▲◊
Rabeprazole	8	0.20 ± 0.09#

Notes: *∗P*<0.01, compared with the control group; ▲*P*<0.01 and #*P* > 0.05, compared with the model group; ◊*P*<0.05, compared with Rabeprazole group.

**Table 7:** IOD values of SP immunoreactive products in the esophageal mucosa in lower third of esophagus of rats in each group $\left(\overset{-}{x}\pm s\right)$.

Group	n	IOD
Control	8	1245.15 ± 1319.97
Model	8	5097.90 ± 2976.49*∗*
NS	8	4087.85 ± 1174.44#
SGHWG	8	1298.36 ± 1233.05▲◊
Rabeprazole	8	3233.47 ± 1692.53▲

Notes: *∗P*<0.01, compared with the control group; ▲*P*<0.05 and #*P* > 0.05, compared with the model group; ◊*P*<0.05, compared with Rabeprazole group.

## Data Availability

The data used to support the findings of this study are included within the article.

## Abbreviations

SGHWG: Shugan Hewei Granule
NS: Normal saline
ACC: Anterior cingulate cortex
NERD: Nonerosive reflux disease
CRF: Corticotropin releasing factor
NMDA-R: N-methyl-D-aspartic acid receptor
SP: Substance P
GERD: Gastroesophageal reflux disease
PPI: Proton pump inhibitor
IOD: Integrated optical density
AOD: Average optical density
EE: Erosive esophagitis
SCL: Symptom checklist
fMRI: Functional magnetic resonance imaging
PVN: Paraventricular nucleus
HPA: Hypothalamic pituitary adrenal axis
IBS: Irritable bowel syndrome.
